# Rad-Bio-App: a discovery environment for biologists to explore spaceflight-related radiation exposures

**DOI:** 10.1038/s41526-021-00143-x

**Published:** 2021-05-11

**Authors:** Richard Barker, Sylvain V. Costes, Jack Miller, Samrawit G. Gebre, Jonathan Lombardino, Simon Gilroy

**Affiliations:** 1 Department of Botany, University of Wisconsin-Madison, Madison, WI USA; 2 Space Biosciences Division, NASA Ames Research Center, Mountain View, CA USA; 3 Lawrence Berkeley National Laboratory, Berkeley, CA USA; 4 Wyle Labs, NASA Ames Research Center, Mountain View, CA USA; 5 Microbiology Doctoral Training Program, University of Wisconsin-Madison, Microbial Sciences Building, Madison, WI USA

**Keywords:** Databases, Physiology, Plant sciences, Microbiology, Molecular biology

## Abstract

In addition to microgravity, spaceflight simultaneously exposes biology to a suite of other stimuli. For example, in space, organisms experience ionizing radiation environments that significantly differ in both quality and quantity from those normally experienced on Earth. However, data on radiation exposure during space missions is often complex to access and to understand, limiting progress towards defining how radiation affects organisms against the unique background of spaceflight. To help address this challenge, we have developed the Rad-Bio-App. This web-accessible database imports radiation metadata from experiments archived in NASA’s GeneLab data repository, and then allows the user to explore these experiments both in the context of their radiation exposure and through their other metadata and results. Rad-Bio-App provides an easy-to-use, graphically-driven environment to enable both radiation biologists and non-specialist researchers to visualize, and understand the impact of ionizing radiation on various biological systems in the context of spaceflight.

## Introduction

**Fig. 1:**
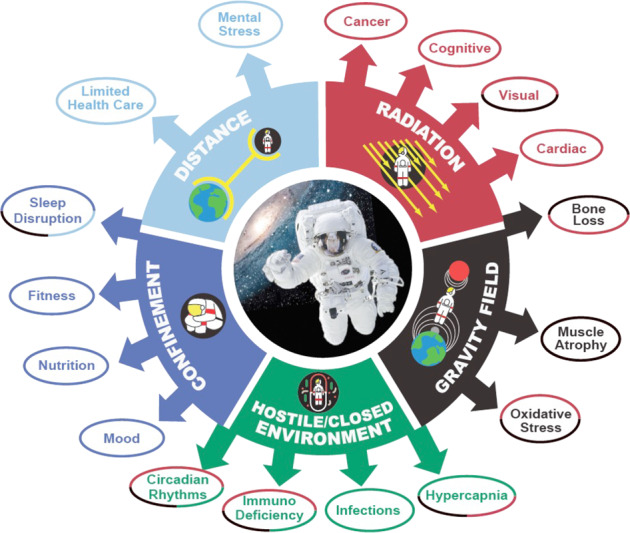
Physical and biological factors affecting human space exploration and their associated stressors including isolation, long confinement in closed environments, microgravity, and ionizing radiation. These factors are likely to act both alone and in combination to impose effects on the crew in space.

The space environment introduces organisms to a unique range of challenges. For example, for humans the closed environment of space vehicles alongside enforced confinement, and the isolation inherent in the vast distances traveled all have important impacts on the functioning of the crew’s physiology and psychology (Fig. 1). Many of these effects are being explored in Earth-based analogs, such as the Hawaii Space Analog and Simulation and the Scientific International Research in Unique Terrestrial Station missions^1,2^. However, some features of space are complex or impossible to simulate on the Earth’s surface, requiring exploration through the relatively rare opportunity of a spaceflight experiment. For example, exposure to sustained microgravity and to the increased ionizing radiation in space are both beyond the experience of terrestrial biology, and currently almost impossible to mimic on the Earth’s surface. Thus, their individual and combined effects on biology remain poorly understood, yet these are likely to be critical factors in how organisms respond to living in space.

A combination of the shielding effects of the atmosphere and the ability of the magnetosphere to deflect radiation around the planet means, that for most of Earth’s history its organisms have been relatively protected from space radiation. Although terrestrial life does encounter natural background radiation from geological sources and UV radiation from the Sun, it is low by comparison to space, with an average annual dose equivalent per person in the US of around 6 mSv^3^, depending on altitude and soil composition (e.g., the presence of minerals that produce radon gas). In deep space, the radiation exposures will be much higher and include highly ionizing single particles not normally found on Earth. Based on data from the Radiation Assessment Detector (RAD) on the Mars Science Laboratory (MSL) during its cruise phase from Earth to Mars and the measurements it made on the Martian surface, the crew on a ~1000 day mission to Mars, assuming “quiet” periods of solar activity throughout, would receive ~360 mGy absorbed dose, much of it from highly ionizing galactic cosmic ray (GCR) ions^4,5^. This is similar to several decades of absorbed background dose on Earth, but as noted, from a very different spectrum of radiation and delivered at a very different dose rate. All of these factors are likely to play into the precise effects this radiation exposure will have on the crew of such a mission, making it complex to extrapolate from terrestrial models to the spaceflight environment.

Indeed, the effects of exposure to space radiation have been shown to induce more deleterious effects in tissues than similar doses of ionizing radiation on Earth, with, e.g., as much as 40 times more biological effectiveness to induce Harderian gland cancers in mice^6^. However, in addition to inducing cancer, radiation exposure in mammals can lead to a wide array of cellular and microenvironment effects such as chronic inflammation, tissue degeneration and impaired nerve function (e.g., refs. ^7,8^). Indeed, all organisms are subject to radiation effects, with animals, microbes, and plants experiencing a variety of radiation-induced cellular events ranging from DNA damage and oxidative stress to shifts in metabolism^9,10^. Different species exhibit varying degrees of radiation tolerance and within a species radiation response is modulated by an individual’s genetics^11–14^. For example, bacteria in the genus Deinococcus can survive radiation doses far in excess of those that would be lethal in humans^15^. Predicting such effects are further confounded by the influence of the spaceflight environment, such as living in microgravity, which itself modulates an organism’s radiation response (e.g., refs. ^16–18^). These complexities in potential biological effects coupled with the relatively limited opportunities to perform direct spaceflight experiments make assessing the risks of radiation exposure to crew members on extended, deep space missions extremely difficult^19,20^. Yet, as the duration and distances of spaceflight missions increase, the question of how different organisms respond to the effects of increased space radiation exposure and the potential for interactions with, e.g., the microgravity of spaceflight becomes increasingly important to address.

To date, much biology-focused spaceflight experimentation has been published without its associated radiation data and though radiation exposure has usually been recorded, it is often not readily accessible. This gap has been recognized by the NASA GeneLab project, which acts as a repository for genomic, transcriptomic, proteomic, and metabolomic data from experiments flown in space or exposed to simulated space stressors^21–23^. Alongside the raw omics datasets, the GeneLab data repository organizes associated metadata into a publicly accessible database called the GeneLab Data System (GLDS^21,22^). Mining of GeneLab’s omics data then enables exploration of the network of molecular responses to space environments as a function of experimental factors described in the metadata. GeneLab has now aggregated much of the radiation metadata associated with its spaceflight-related datasets^24^. In other words, the GLDS now provides the raw materials of data and metadata for making broad inferences about radiation effects across multiple sets of spaceflight experiments and in different organisms. The GeneLab Application Programming Interface (API) also enables access to these data by external web applications, with the aim of allowing the scientific community to deploy custom tools to visualize and explore these data in ways complementary to the capabilities available in GeneLab’s own data visualization portal.

In the work presented here, we introduce one such custom visualization tool, the Rad-Bio-App (https://astrobiology.botany.wisc.edu/astro-rad-bio-app). Rad-Bio-App capitalizes on the “FAIRness” standards and rich data curation of the GLDS^25^ to provide an interactive graphical interface through which investigators can visualize, filter, and compare radiation dosimetry and other metadata across the GLDS. The reiterative filtering capabilities of Rad-Bio-App further allow the user to define datasets with potentially common radiation effects, or to explore interactions between radiation exposure and a variety of space-related experimental conditions. These datasets then provide targets for further in-depth analyses using a variety of bioinformatics-related tools.

### Overall design and goals of the Rad-Bio-App

**Fig. 2:**
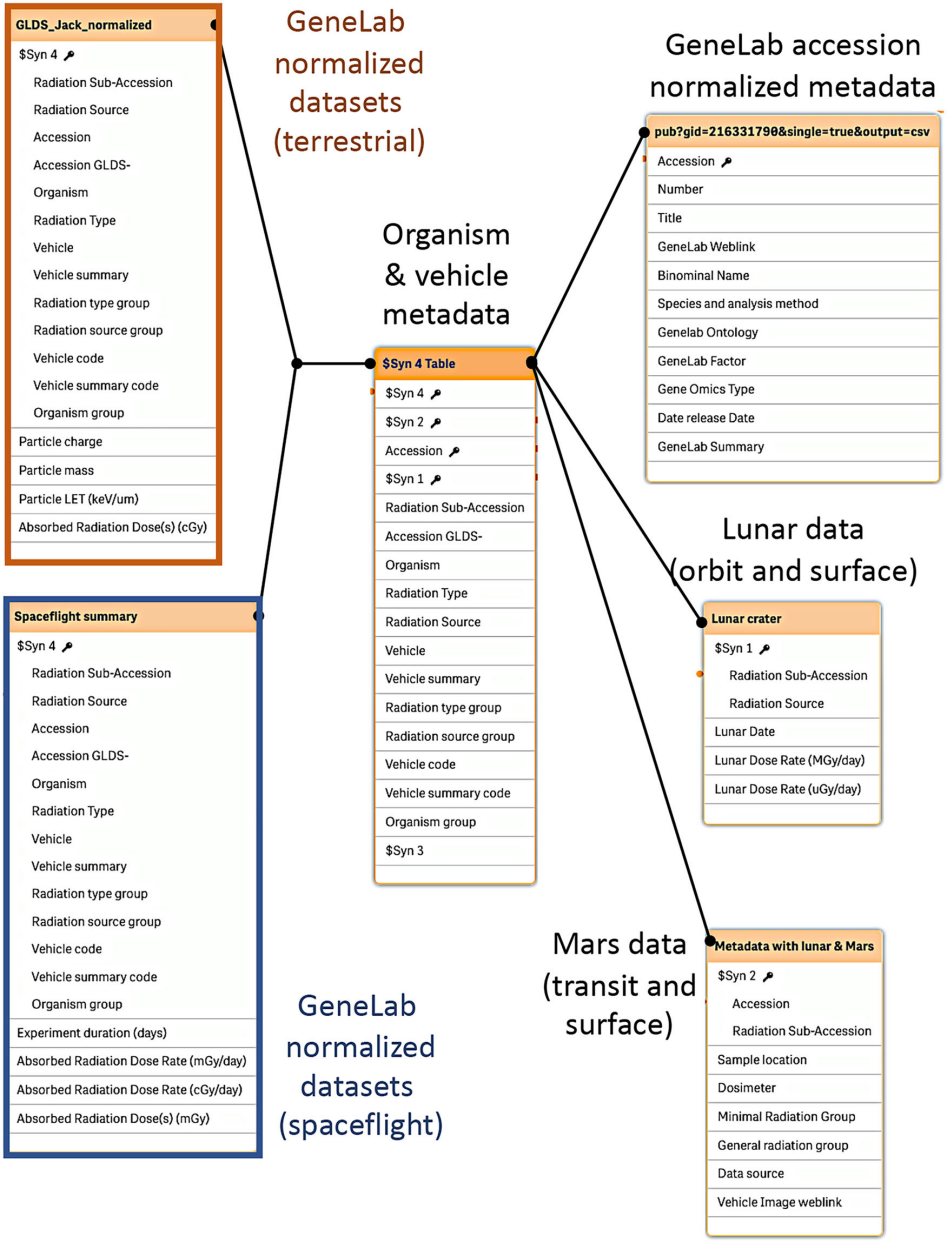
Rad-Bio-App’s data structure. Rad-Bio-App imports datasets from ground-based space radiation simulations, spaceflight experiments, the RAD detector of the Curiosity Rover, the CRaTER instrument on the lunar Reconnaissance Orbiter, the LRD of the Chang’E 4 lunar lander, the RaD-X high altitude balloon and dosimetry from Apollo missions. These datasets are linked in a relational database within the Qlik data management system. At the time of writing, the App contains data from 132 accessions, each with associated metadata including: Accession #, GeneLab GLDS-Accession #, Radiation Sub-Accession (individual GLDS accessions contain data from multiple treatments, which we define as sub-accessions and denote as GLDS#.#, e.g., GLDS-93.1, GLDS-93.2 etc.), organism, analysis type (trancriptomics, genomics, metabolomics etc.), radiation type, particle charge, particle mass, radiation source, particle LET (keV/µm), energy (MeV classified to ions, gamma-rays, or x-rays), minimal radiation group, total absorbed radiation dose (Gy), summary dose rate (Gy/day), exposure duration, general radiation group, vehicle (for spaceflight experiments), sample location, (for spaceflight experiments), and dosimeter type.

The overall goal of the Rad-Bio-App is to allow users to develop filtered, curated lists of omics datasets within the GeneLab data repository, that can then be explored for predicted radiation-related responses. Therefore, documenting the origin of such data is of paramount importance and is central to the Rad-Bio-App’s data structure outlined in Fig. 2. Sources of these spaceflight datasets include experiments performed on the Space Shuttle, the International Space Station (ISS), satellites (including the Russian Bion and Foton) and the Chinese Shenzhou spacecraft. Ground data are typically from experiments that use terrestrial radiation sources such as particle accelerators and gamma, x-ray, and neutron sources. We have also incorporated summary information from high altitude balloons on Earth^26^ and orbital and surface data from the Moon, from the journey from Earth to Mars and from the Martian surface by importing data from the Cosmic Ray Telescope for the Effects of Radiation (CRaTER) instrument on the Lunar Reconnaissance Orbiter (LRO)^27^, the RAD instrument of the Mars Science Laboratory^4,5,28^, the Lunar Lander Neutrons and Dosimetry (LND) experiment mounted on the Chang’E 4 lunar lander^29–31^ and aggregate data from the Apollo astronauts’ Radiation Survey Meters (RSM)^32^. CRaTER provides data on radiation in lunar orbit and LND for the lunar surface. The RAD measured radiation exposure while both in transit to Mars and on the Martian surface. Supplementary Table 1 presents a summary of these balloon, lunar and Mars-related data.

When combined with the linked GeneLab metadata of features such as species, hardware, and radiation type, this data architecture allows the user to explore, filter, and compare the radiation environments experienced across many different types of space biology studies and even those of likely future missions. The resulting lists of GLDS datasets selected for specific characteristics of radiation exposure, then offer the possibility of further interrogating the associated omics data within GeneLab, employing either GeneLab’s visualization portal tools or other custom techniques (e.g., for plants, exploration within the TOAST database^33^). Such approaches allow the researcher to search for molecular fingerprints between experiments that are associated with common or different radiation exposures, and also ask how other factors within the experiment may be impacting these responses. The Rad-Bio-App should therefore help the scientific community both explore how biology responds to ionizing radiation exposure in the context of spaceflight, and also help identify space radiation conditions currently not being simulated on the ground.

**Fig. 3:**
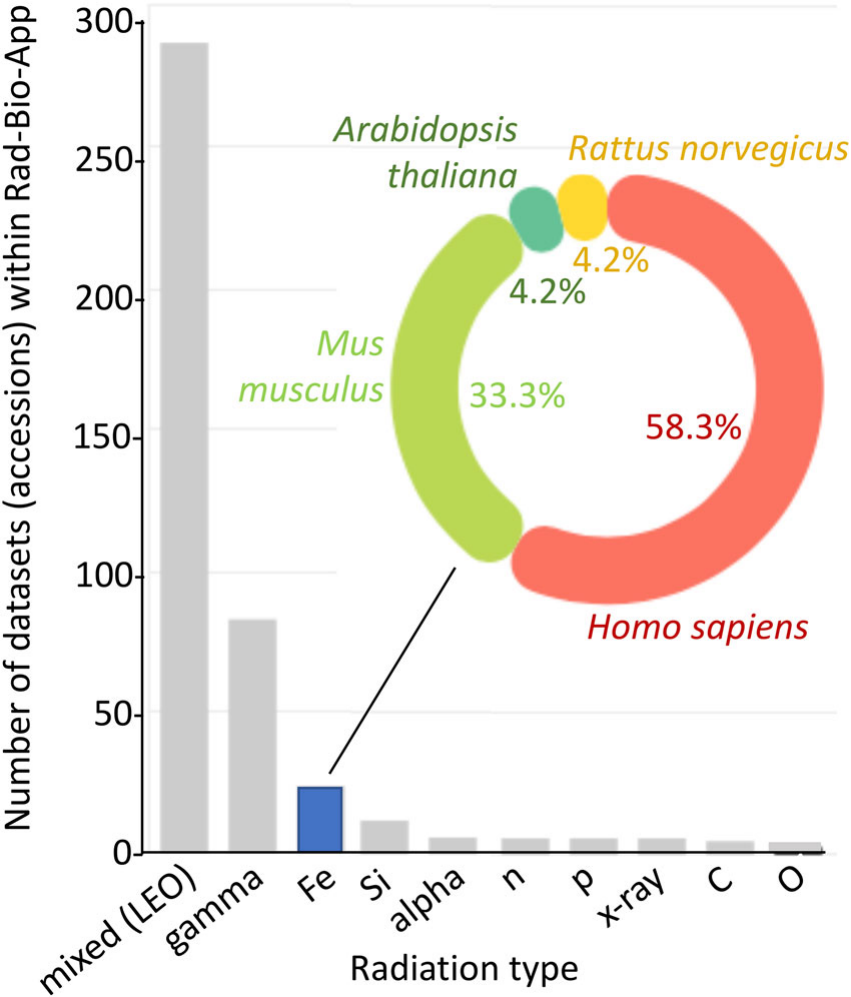
Datasets for terrestrial experiments exposing biology to different radiation types that are deposited at GeneLab and cross-referenced within the Rad-Bio-App. Shown inset is the distribution of species for the Fe datasets. Mixed LEO represents the varied array of spaceflight data. Output extracted from elements of Rad-Bio-App’s graphical interface.

It is important to note here that within the data visualization portal on the GeneLab website users can search for omics datasets related to specific radiation metadata classes: radiation type, energy, dose, and dose rate. Rad-Bio-App complements these capabilities by extending the range and details of the searchable radiation metadata, and providing an interactive ability to apply quantitative filters simultaneously across all datasets. To give one example of the utility of implementing such extended capabilities: for operational reasons related to the available radiation source, there is a great deal of data from ground-based studies on a number of different organisms exposed to iron ions at the NASA Space Radiation Laboratory (NSRL) at Brookhaven National Laboratory (Fig. 3). Thus, these datasets potentially provide a wealth of insight by comparing experiments with similar irradiation characteristics. A user who is expert in this area would know to search for this type of radiation exposure to aggregate this extensive set of results, a task addressable through GeneLab’s visualization portal. Using the Rad-Bio-App, a biologist relatively new to the space radiation field would quickly identify iron as a radiation type that is rich in datasets through simple exploration of the graphical representation of the aggregate terrestrial radiation data (Fig. 3). Further, they could rapidly define whether their species of interest lie within these datasets. Thus, Rad-Bio-App should also help to broaden access and usage of these kinds of data^23^ by allowing researchers who do not specialize in radiation biology to begin to incorporate potential radiation effects into their analyses and experimental designs.

### The characteristics of the radiation data within Rad-Bio-App

Space radiation comprises a mix of particles and energies arising from the Sun and the background galactic cosmic radiation (GCR) that originates outside of the Solar System^34^. Therefore, Rad-Bio-App provides filtering tools to help navigate some of these intricacies. Solar particles consist of an intense flux of mostly protons and electrons with energies up to about 100 keV, but sporadic solar particle events can accelerate these particles to higher energies (about 1–10 MeV^35^). GCR consists of protons and heavier ions ejected from supernovae and can be even more energetic (above 100 MeV/nucleon). Nuclear or electromagnetic interactions of such primary radiation with other materials, such as the hull or shielding within a spacecraft can also lead the production of secondary particles. These secondary particles include protons, neutrons, and light charged fragments, which can themselves be highly penetrating and, as for solar particles and GCR, biologically damaging^6^. To help address this complexity in the spectrum of the types of radiation that may be present in an experiment, the Rad-Bio-App allows filtering of the data for specific energies and doses and, for the terrestrial studies with well-defined radiation sources, by the radiation quality (e.g., alpha, Fe nucleus, proton, etc.).

In relation to effects on biological systems, a further key characteristic of radiation is the energy it can deliver over a distance traveled (its Linear Energy Transfer, or LET, typically expressed in kiloelectron volts per micrometer; keV/µm). Low LET radiation is loosely defined to consist of particles with <10 keV/μm, such as protons and electrons, whereas high LET radiation is composed of energetic nuclei with greater penetrating power and consequently a greater potential to damage biological molecules^36^. However, even low LET radiation can still readily disrupt biological processes through effects such as DNA damage and oxidative stress^37,38^. Therefore, the Rad-Bio-App allows filtering on the LET of the radiation quality when known.

### The dose vs. dose rate challenge

One other major challenge in studying the effects of space radiation on biology is the difficulty of simulating the spectrum of space radiation conditions in the laboratory, whether on the ground or in Low Earth Orbit (LEO). This issue makes it important to place the inferences a researcher can draw from Rad-Bio-App-based analyses in this context. Particle accelerator facilities on Earth, such as the NSRL, can provide low absolute doses to try to mimic expected radiation exposure in space but generally these have to be delivered in an acute manner, i.e., at dose rates on the order of Gy/min, compared to the typical rates of hundreds of µGy/day that are experienced in Space. In addition, until recently, exposures in these ground-based experiments were limited to one particle type at one energy at a time, whereas, as discussed above, space radiation represents a complex mix of radiation types. Rad-Bio-App provides filters for dose rate and particle types, when known, to help place these space radiation analog experiments in context. Fortunately, ground facilities, while still limited to relatively high dose rates, are developing more sophisticated mixed field capabilities that promise to more closely mimic the spectrum of particles eliciting space radiation effects. However, it remains a significant challenge to perform faithful space radiation-related analog experiments on the ground, and so it is critical to remember these issues when conducting Rad-Bio-App analyses.

Orbiting spacecraft offer dose rates more appropriate for understanding space-related effects on biology, but again when using the Rad-Bio-App for comparisons between datasets it is important to be aware of the effects of the locale on the radiation environment. Thus, although particle types are generally not recorded for experiments conducted in spaceflight (Rad-Bio-App classes these as “Mixed” radiation), the effects of shielding from the magnetosphere means that radiation exposures in LEO will likely have a much higher proton contribution than will exploration missions traveling to the Moon and beyond. Future missions such as Artemis^39^, Lunar Gateway^40^, and BioSentinel^41^ promise to provide data from biology operating well beyond these confounding effects of LEO. Both the GeneLab data system and Bio-Rad-App are designed to readily incorporate information from these new facilities and missions as they become available to help more faithfully align future analyses to the range of likely space radiation profiles.

### Architecture of the Rad-Bio-App exploration environment

Rad-Bio-App is built using the Qlik database management software (Qlik Technologies Inc., King of Prussia, PA, USA). Radiation data have been aggregated from NASA’s GeneLab data repository^21,23^ and data recorded by the RaD-X high-altitude balloon mission, CRaTER, the RAD, the LRD and on Apollo missions. These datasets and associated metadata were then manually curated and linked through the Qlik software engine to provide a searchable relational database (Fig. 2). Qlik provides customizable interactive representations of datasets, allowing for their presentation as a simple graphical user interface built around data-centric dashboards. Data filters are incorporated allowing the user to rapidly narrow searches and comparisons by e.g., organism, radiation type, or mission and these filters then spawn to all other datasets and analysis dashboards, allowing the user to reiteratively narrow their search and comparisons across the breadth of all the data within Rad-Bio-App as described below.

### Navigating the Rad-Bio-App: dashboards

**Fig. 4:**
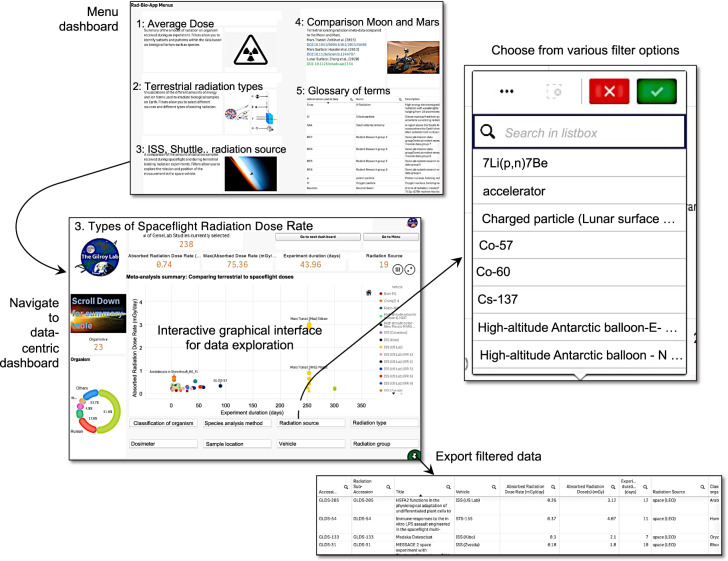
Navigation within Rad-Bio-App is through dashboards accessed from a central menu page. Each data exploration dashboard allows for reiterative, interactive filtering on factors such as radiation type, mission, and spaceflight hardware and allows for both dynamic visualization of the filter effects and for export of the filtered data to a spreadsheet.

Rad-Bio-App is navigated through an initial menu or index page that presents icons and brief descriptions linking to additional pages or dashboards, as shown in Fig. 4. Each dashboard simplifies the access to a particular kind of radiation data along with the visualization and filtering tools to be able to explore those datasets.

*Dashboard #1* lets the researcher find datasets by radiation exposure level. Users land on a summary of the average radiation exposure each organism experienced during an experiment indexed to the relevant dataset within the GeneLab data repository. These radiation data can be further filtered on metadata factors such as radiation type, radiation source, and exposure levels.

*Dashboard #2* lets the user find datasets by the type of radiation from different sources. One will notice in this dashboard that a large number of datasets are classified as LEO experiments (e.g., ISS, Space Shuttle), whereas radiation data related to terrestrial experimentation allows, e.g., selecting the particle of interest (e.g., 1 GeV/n Fe). The Rad-Bio-App provides additional filtering of these datasets on features such as organism and the analytical approach used to assess biological response (e.g., transcriptomics, genomics, and proteomics).

*Dashboard #3* lets the user focus on experiments conducted in space, which typically have much lower dose rates than experiments on Earth and reflect chronic exposure lasting days to weeks. As such, the dashboard lands on a graph displaying radiation doses as a function of duration. One can sub-select these spaceflight-related experiments by filtering for, e.g., mission, dosimeter, and flight hardware. It is important to note that, unlike terrestrial radiation experiments that use defined radiation sources, the precise kinds of particles responsible for the radiation exposure in most spaceflight experiments are not measured and so, as noted above, they are grouped collectively as “Mixed” radiation exposures on this dashboard. Therefore, the filter for radiation source or group in dashboard #3 is based on the region of space, where the measurement was taken (such as LEO) rather than specific radiation type as available for the terrestrial studies in the previous dashboard.

*Dashboard #4* provides an example of a comparative analysis between maximum, minimum and average lunar orbit, lunar surface, Martian surface, and Earth-to-Mars transit radiation doses versus the wealth of spaceflight and terrestrial biological experiments available at NASA’s GeneLab data repository. The long duration of data capture for these Moon-space and Mars-space measurements means that elements such as intense solar particle events, when the Sun emits extremely high levels of radiation for a short duration, can impact on average measurements. Therefore, in addition to maximum and minimum exposures, Rad-Bio-App presents several “average” exposure rates for the lunar and Martian datasets. These averages include both values with high intensity stochastic events included and measurements drawn from periods, when these events did not occur (summarized in Supplementary Table 1). For the Martian data, we also present average measurements from the RAD’s Si detector and the Si detector data corrected for radiation penetration into water. This latter measurement provides a closer analog of how the radiation is likely interacting with biology than the Si detector’s raw output. This correction relies upon a calculation from the Si detector’s measurement using a dose conversion factor, and so should be taken as an approximation^5^.

*Glossary Dashboard:* Finally, the App also presents a glossary of terms on the main dashboard index page and mousing over this table reveals a full screen expansion icon, allowing the glossary to be seen on a single browser page. The homepage of the Rad-Bio-App also links to a video tutorial, which leads potential users through accessing some of the basic functions of the App.

### Test case: understanding radiation biology on the lunar surface

**Fig. 5:**
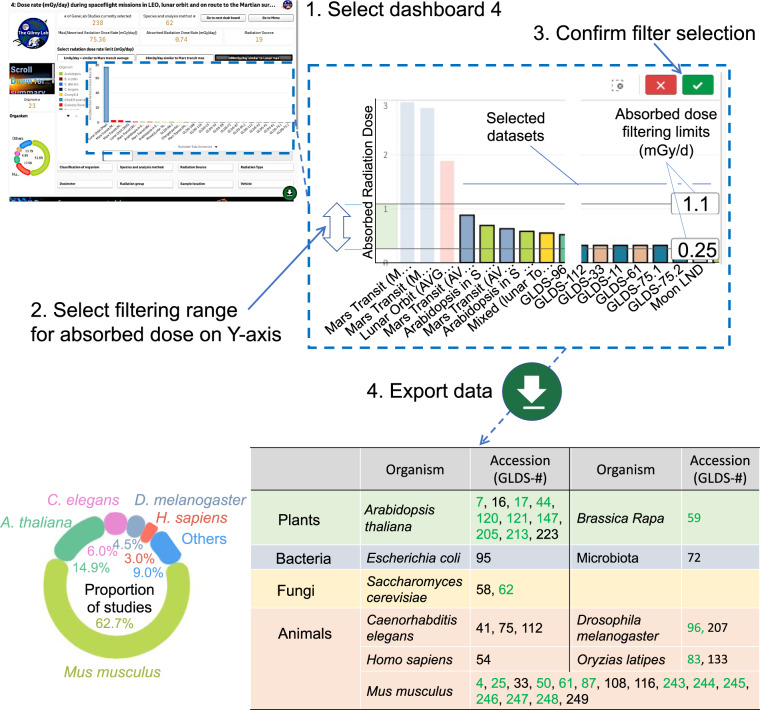
Example of use of Rad-Bio-App to define datasets within the GeneLab data repository mimicking a lunar surface exposure. The data within dashboard #4 of the Rad-Bio-App contains radiation data from Martian, lunar and spaceflight missions and ground-based radiation experiments. These data were filtered using radiation levels recorded on the lunar surface by the Chang’e 4 lander, with limits set to span a 2-fold range around the average dose rate (0.55 mGy/d). Results of this filtering were then exported to build a listing of spaceflight experiment datasets within the GeneLab data repository where their radiation exposure lies within this range of dose rates. Green numbers in table, datasets pre-processed by either the GeneLab uniform microarray or RNAseq analysis pipelines.

**Fig. 6:**
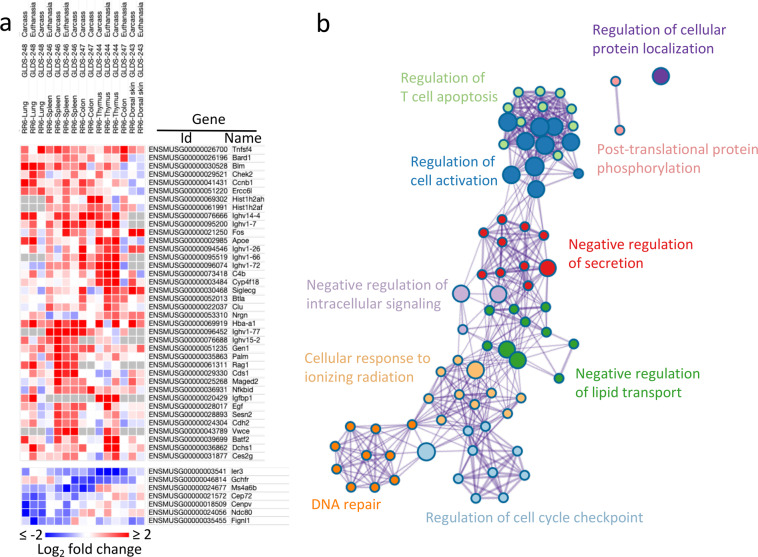
Analysis of radiation responsive mouse genes showing altered expression in spaceflight. **a** Log_2_-fold change of differentially expressed genes (flight vs ground or flight vs. basal) from mouse RNAseq datasets identified from Fig. 5 (GLDS 243, 244, 246, 247, 248) were cross-referenced to genes annotated as being radiation responsive in the RadAtlas database^50^. **b** Ontology enrichment analysis performed using Metascape^51^. Ground control is sample harvested synchronously with flight samples, basal control are samples harvested immediately prior to flight.

The Rad-Bio-App is designed to allow researchers rapid access to data on the radiation environment of a particular study and to make easy comparisons across datasets to reveal new insight. As an example, we have used Rad-Bio-App to explore possible effects of the radiation in the lunar environment. Thus, as the sights of the spaceflight community begin to widen to include crewed missions to the Moon and eventually Mars, it becomes crucial to ask how radiation will affect biology under these conditions. However, to date, with the exception of the 27 Apollo astronauts who travelled to the Moon and the short duration of the seedling germination experiment in China’s Chang’E-4 mission^29,30^, we have no data on the functioning of biology in the lunar environment. How then to make predictions about the radiation effects to inform the future missions to this destination? Rad-Bio-App incorporates direct radiation measurements made by CRaTER as it orbits the Moon, from the Chang’E-4 lander on the lunar surface and aggregate data from astronaut dosimetry on the Apollo missions. Thus, Rad-Bio-App allows filtering the array of LEO spaceflight and terrestrial data from NASA’s Genelab repository for radiation exposures lying close those expected for the lunar environs. An example of this kind of analysis is shown in Figs. 5 and 6, using Dashboard #4 of the Rad-Bio-App. For this analysis, we explored the lunar surface environment and so used the direct measurements of lunar surface radiation made by Chang’E 4 to ask, if there were datasets within the GeneLab data repository that might help inform on how biology would act under these radiation conditions. Briefly, we took the average mixed lunar surface radiation dose rate from the LRD detector on Chang’E-4 (0.55 mGy/day) and set limits of 2x − 0.5x (i.e., searching for <1.1 to >0.25 mGy/day—see Fig. 5 step 2), assuming it would broadly cover a likely lunar radiation dose rate. This approach identified 32 experiments (Fig. 5, step 3), generating a table at the bottom of the dashboard which we downloaded by clicking on the download icon (down green arrow in Fig. 5, step 4). Rad-Bio-App further showed that the corresponding datasets span ten different organisms. Here, we capitalized on the synergy possible between the well-curated data repository at GeneLab and the rapid dataset surveys possible through Rad-Bio-App. Thus, interrogating the GeneLab data repository revealed gene expression analysis was performed on flight samples versus a paired ground control in most of the studies identified in the above analysis. Rad-Bio-App also readily showed that the majority (15) of these studies were from mice, with analyses of whole genome gene expression performed using either microarray or RNAseq technologies. In addition, cross-referencing to the GeneLab visualization portal revealed that most of these datasets have been processed through GeneLab’s uniform microarray or RNAseq software pipelines (Overbey et al.^42^). That is, these datasets have been pre-processed within the GeneLab data system using the same computational approaches, and the analyzed results on differential gene expression deposited and made publicly available for download.

Briefly, for these analytical pipelines, the GeneLab analyses were implemented in the Galaxy software environment^43^, with the microarray analysis using the R/Bioconductor software package Limma^44^ and RNA-seq using STAR (v2.7.1a^45^) and RSEM (v1.3.1^46^). Further descriptions of the processing and links to the analytical codes used, along with the deposited analyzed datasets are available on the Genelab website^22^. Application of the same analytical steps for each dataset allows for much more robust comparisons between studies, and the free access to the deposited results greatly speeds and simplifies subsequent exploration.

These analyses then provided us with target datasets within the GeneLab data repository to explore for common molecular signatures from biology experiencing radiation dose rates similar to what we might predict on future missions to the Moon. One must note here that comparing microarray and RNAseq datasets can be complex to perform and interpret due to potential artifacts generated by mixing results from these different analytical technologies (e.g., ref. ^47^). Therefore, to increase the robustness of our further analyses, we focused on five RNAseq datasets derived from the same mission: Rodent Research 6 (RR6)^48^. These RR6 datasets were from five different tissues: dorsal skin, thymus, spleen, colon, and lung, respectively (i.e., GLDS 243, 244, 246, 247, and 248) and they were processed using the GeneLab RNAseq pipeline^42^. These datasets had multiple controls: tissues were either collected from sacrificed animals and then preserved (i.e., euthanasia), versus where the whole animal was first frozen (to be dissected later by thawing) at the same time as carcasses preserved in space by astronauts (i.e., carcass). Another factor differentiating various controls were animals sacrificed just before the mission (i.e., basal control) versus animals sacrificed on the ground at the same time as in space (i.e., ground control—GC). Such experimental factors were recently described in detail and their impact on gene expression discussed^49^. These five different tissues provided ten flight-to-basal-control and five flight-to-GC comparisons within the pre-processed datasets deposited at GeneLab, and so we downloaded the gene expression data for these 15 comparisons from the GeneLab data repository for further analysis.

We next interrogated the RadAtlas^50^, a tool that provides an aggregation of gene expression profiles of radiation-associated and radiation-responsive genes drawn from the ground-based literature. We downloaded the aggregated data from the RadAtlas and asked, which of the genes identified as transcriptionally responsive to radiation in that tool also showed transcriptional changes in the mouse spaceflight RNAseq datasets described above (Supplementary Data 1). We then filtered this list to ask, of these genes, which of them showed agreement in either induction or repression in spaceflight to greater than 2-fold in at least four of the mouse datasets. To increase the rigor of this filtering, we set the further criterion that no dataset showed a conflicting response. i.e., if four or more datasets showed induction of a particular gene, none of the other datasets also showed repression at the 2-fold cutoff (Fig. 6a and Supplementary Data 1). This thresholding allowed us to identify putative shared radiation response genes whilst filtering out those showing small or inconsistent responses. We then used the gene network annotation and analysis tool Metascape^51^ to visualize the functional groupings (ontologies) associated with these sets of genes (Fig. 6b). This list showed enrichment in features ranging from T-cell apoptosis, to DNA repair and the regulation of the cell cycle, providing targets for future exploration of possible radiation effects in the spaceflight environment through e.g., targeted flight experimentation.

## Conclusions

To conclude, we have introduced a data mining visualization tool, the Rad-Bio-App, enabling the rapid identification of omics datasets deposited in the NASA GeneLab data repository, which are relevant for a specific spaceflight radiation scenario (e.g., trip to Mars or on the lunar surface) or specific characteristics of radiation exposure. Testing this tool on a scenario of a mission on the Moon, we identified a series of experiments conducted on the ISS with similar radiation profiles. Because the Rad-Bio-App links to GeneLab datasets, it was then straightforward to extract processed gene expression tables from GeneLab to identify common pathways that may be modified by, in this case, the lunar surface radiation environment. As more researchers enter datasets into GeneLab related to spaceflight and radiation exposure that explore the effects of e.g., species, genetics, gender, and age, the Rad-Bio-App should become an increasingly powerful tool to help accelerate assessment of the effects of Space radiation environments on living entities.

## Supplementary information

Supplementary Table 1

Supplementary Data 1

## Data Availability

Rad-Bio-App is accessible at: https://astrobiology.botany.wisc.edu/astro-rad-bio-app. The data analyzed in the Rad-Bio-App are available at the GeneLab data repository: https://genelab-data.ndc.nasa.gov/genelab/projects/ or from the sites described in the text and tables. The radiation data in the GLDS are from a variety of sources, including the open literature, private communications from investigators and a database maintained by the Space Radiation Analysis Group (SRAG) at NASA-Johnson Space Center (https://srag.jsc.nasa.gov/). Use of data from SRAG in publications or presentations requires consultation with SRAG for data integrity and interpretation and acknowledgement of subject matter expert(s). See the GLDS Environment Data section on the GeneLab website for further details.
