# Supplementary material for: Rad-Bio-App: a discovery environment for biologists to explore spaceflight-related radiation exposures
Source: NPJ Microgravity. 2021 May 11;7:15. doi: 10.1038/s41526-021-00143-x (PMC8113475; doi:10.1038/s41526-021-00143-x)
Supplement: Supplementary file 1 — Supplementary Table 1 [file 41526_2021_143_MOESM1_ESM.pdf]

Accession	Dose Rate (mGy/day)	Dosimeter	Reference	Description
APOLLO (AVG)	0.43	RSM	1	Average for Apollo astronauts' personal dosimeters
Lunar Orbit (AVG 2010-2020)	0.197	CRaTER	2	Includes high intensity stochastic events
Lunar Orbit (Min 2010-2020)	0.189	CRaTER	2	
Lunar Orbit (AVG 2009)	0.204	CRaTER	3	Excludes high energy stochastic events
Lunar Orbit (AVG 2013)	0.372	CRaTER	4	Excludes high energy stochastic events
Lunar Orbit (AVG 2018)	0.195	CRaTER	3	Excludes high energy stochastic events
Lunar Surface (Charged particle)	0.245	LND	5	
Lunar Surface (Total)	0.318	LND	5	
Lunar Surface (Neutral particle)	0.074	LND	5	
Mars Surface Silicon (AVG)	0.209	RAD (Si)	6	Excludes high energy stochastic event
Mars Surface Silicon (Max)	0.227	RAD (Si)	6	Excludes high energy stochastic event
Mars Surface Silicon (Min)	0.178	RAD (Si)	6	
Mars Transit Silicon in water (AVG)	0.481	RAD (Si)	7	Si detector value corrected for penetration in water, excludes stochastic high energy events
Mars Transit (AVG) Plastic	0.881	RAD (plastic)	7	Average extracted from data in Zeitlin et al (2013), includes high energy stochastic events
Mars Transit (AVG) Silicon	0.63	RAD (Si)	7	Average extracted from data in Zeitlin et al (2013), includes high energy stochastic events
Mars Transit (Max) Plastic	2.977	RAD (plastic)	7	Includes high intensity stochastic events
Mars Transit (Max) Silicon	2.872	RAD (Si)	7	Includes high intensity stochastic events
Mars Transit (Min) Plastic	0.411	RAD (plastic)	7	
Mars Transit (Min) Silicon	0.092	RAD (Si)	7	
Mars Transit Plastic (AVG) stochastic events removed	0.461	RAD (plastic)	7	Excludes high intensity stochastic events
Mars Transit Silicon (AVG) stochastic events removed	0.33	RAD (Si)	7	Excludes high intensity stochastic events
Stratosphere balloon (AVG)	0.064	TID, Liulin, RaySure, TEPC	8	Average value multiple dosimeters

Supplementary Table 1. Mars-, Moon- and high-altitude balloon-related data imported into the Rad-Bio-App. AVG, average dose rate over time; CRaTER, Cosmic Ray Telescope for the Effects of Radiation instrument on the Lunar Reconnaissance Orbiter; Liulin, dosimeter-spectrometer from the Bulgarian Academy of Sciences; LND, Lunar Lander Neutrons and Dosimetry mounted on the Chang'E 4 lunar lander; RAD, Radiation Assessment Detector on the Mars Science Laboratory with either a silicon (Si) or plastic (plastic) detector; RaySure, detector provided by the University of Surrey; RSM, radiation survey meter; TEPC, tissue equivalent proportional counter; TID, total ionizing dose. References: 1. Cucinotta, F. A., Hamada, N. & Little, M. P. No evidence for an increase in circulatory disease mortality in astronauts following space radiation exposures. *Life Sci. Sp. Res.* **10**, 53–56 (2016). 2. CRaTER-web.sr.unh.edu/. 3. Zeitlin, C. *et al.* Update on Galactic Cosmic Ray Integral Flux Measurements in Lunar Orbit With CRaTER. *Sp. Weather* **17**, 1011–1017 (2019). 4. Spence, H. E. *et al.* Relative contributions of galactic cosmic rays and lunar proton 'albedo' to dose and dose rates near the Moon. *Sp. Weather* **11**, 643–650 (2013). 5. Zhang, S. *et al.* First measurements of the radiation dose on the lunar surface. *Sci. Adv.* **6**, (2020). 6. Hassler, D. M. *et al.* Mars' surface radiation environment measured with the Mars science laboratory's curiosity rover. *Science* **353**, 1080–1084 (2014). 7. Zeitlin, C. *et al.* Measurements of energetic particle radiation in transit to Mars on the Mars Science Laboratory. *Science* **340**, 1080–1084 (2013). 8. Mertens, C. J. Overview of the Radiation Dosimetry Experiment (RaD-X) flight mission. *Sp. Weather* **14**, 921–934 (2016).

Supplementary Data 1. Differential expression (fold-change flight vs ground or basal controls) of genes in mouse datasets GLDS 243, 244, 246, 247 and 248 identified as also showing radiation responsive transcription from the RadAtlas database.
